# Age-period-cohort modelling of non-Hodgkin's lymphoma incidence in a French region: a period effect compatible with an environmental exposure

**DOI:** 10.1186/1476-069X-9-47

**Published:** 2010-08-08

**Authors:** Jean-François Viel, Evelyne Fournier, Arlette Danzon

**Affiliations:** 1 CNRS n° 6249 "Chrono-Environment", Faculty of Medicine, Besançon, France; 2 Doubs Cancer Registry, EA 3181 Epithelial Carcinogenesis Research Team, Besançon, France

## Abstract

**Background:**

The incidence of non-Hodgkin's lymphoma (NHL) has risen steadily during the last few decades in all geographic regions covered by cancer registration for reasons that remain unknown. The aims of this study were to assess the relative contributions of age, period and cohort effects to NHL incidence patterns and therefore to provide clues to explain the increasing incidence.

**Methods:**

Population and NHL incidence data were provided for the Doubs region (France) during the 1980-2005 period. NHL counts and person-years were tabulated into one-year classes by age (from 20 to 89) and calendar time period. Age-period-cohort models with parametric smooth functions (natural splines) were fitted to the data by assuming a Poisson distribution for the observed number of NHL cases.

**Results:**

The age-standardised incidence rate increased from 4.7 in 1980 to 11.9 per 100,000 person-years at risk in 1992 (corresponding to a 2.5-fold increase) and stabilised afterwards (11.1 per 100,000 in 2005). Age effects showed a steadily increasing slope up to the age of 80 and levelled off for older ages. Large period curvature effects, both adjusted for cohort effects and non-adjusted (p < 10^-4 ^and p < 10^-5^, respectively), showed departure from linear periodic trends; period effects jumped markedly in 1983 and stabilised in 1992 after a 2.4-fold increase (compared to the 1980 period). In both the age-period-cohort model and the age-cohort model, cohort curvature effects were not statistically significant (p = 0.46 and p = 0.08, respectively).

**Conclusions:**

The increased NHL incidence in the Doubs region is mostly dependent on factors associated with age and calendar periods instead of cohorts. We found evidence for a levelling off in both incidence rates and period effects beginning in 1992. It is unlikely that the changes in classification (which occurred after 1995) and the improvements of diagnostic accuracy could largely account for the 1983-1992 period-effect increase, giving way to an increased exposure to widely distributed risk factors including persistent organic pollutants and pesticides. Continued NHL incidence and careful analysis of period effects are of utmost importance to elucidate the enigmatic epidemiology of NHL.

## Background

The aetiology of the most common forms of non-Hodgkin's lymphoma (NHL) remains elusive [1]. The incidence of NHL, however, has risen steadily in many countries during the second half of the 20th century, making this group of malignancies an increasingly important contributor to the overall cancer burden. This upward trend was observed in all geographic regions covered by cancer registration and was not restricted to any particular age group or gender or to predominantly rural or urban areas [2]. This epidemic of NHL has now begun to level off in North America and Europe [3], with a recent downturn among white males aged 25-54 years in the USA [4] and among males aged 30-39 years in the Nordic countries [5]. In France, incidence trends showed an increase in both genders between 1980 and 2005 (age-standardised incidence rates: 6.2 to 12.1 and 4.0 to 8.2 for males and females, respectively), with a slower rate of increase from 2000 onwards [6]. The interpretation of long-term trends in the incidence of NHL has attracted much speculation in the absence of a clear understanding of the risk factors. The generalised increases in NHL incidence do not appear to be explicable only in terms of better diagnostics or classification [7]. Moreover, it is now clear that AIDS-related NHL accounts for a limited proportion of this increase in developed countries [8]. Thus, most authors agree that the steady increases in NHL rates up to the late 1990s almost certainly reflect real increases in disease incidence [9].

The escalation in NHL incidence suggests increasing exposure to one or more ubiquitous lymphomagenic agents [1] and could therefore be partly explained by environmental exposure to common chemicals (such as persistent organic pollutants or pesticides). One dioxin congener (2,3,7,8-T4CDD or TCDD) and one furan congener (2,3,4,7,8-P5CDF) are considered carcinogenic to humans by the International Agency for Research on Cancer for all cancers combined [10]. For NHL specifically, increased incidence and mortality have been reported in several investigations conducted on cohorts of workers exposed to TCCD [11-14], on the population exposed to the accidental industrial release in Seveso (Italy) [15] and among neighbours of municipal solid waste incinerators [16,17]. Polychlorinated biphenyl (PCB) 126 is also classified as a known human carcinogen [10] and there is mounting evidence of a relationship between PCBs and NHL risk [18-21]. Recent reviews have highlighted pesticide exposure as one of the likely occupational risk factors for NHL [22,23].

Further elucidation of the temporal changes in NHL incidence has important epidemiologic and public health implications [4]. In this context, it is important to understand whether these time trends are related to age, calendar period or birth cohort because these time scales are surrogates or proxy measures for other influences. Although age-period-cohort (APC) models are descriptive tools, the identification of changes in the magnitude of long-term trends can have important aetiological implications [24]. They are therefore likely to further our knowledge of the enigmatic aetiology of NHL [2,25]. More precisely, if the environmental hypothesis holds, it should result in increased period effects (indicative of changes in risk factors that affect all ages equally). Additionally, prenatal exposure to environmental risk factors during a given time period would induce a birth cohort effect.

Cancer registration does not cover the whole French population. In 1992, the situation was particularly unfavourable, as cancer registration covered only 3% of the population and this included specialized cancer registries that collect data on certain types of cancer only [26]. Established in 1976, the Doubs Cancer Registry is the second oldest cancer registry in France. This long tenure provides a unique opportunity to explore temporal changes in French cancer incidence.

The aims of this study were to assess the relative contributions of age, period and cohort effects to NHL incidence patterns and therefore to provide clues to explain the increasing incidence in the French Doubs region.

## Methods

### Study site

The Doubs region (5233 km^2^, 499 162 inhabitants in 1999) is located in eastern France along the Swiss border. Mainly rural, this region is, however, industrialized in its north-eastern part.

### Population data

Population data were available from the French Census Bureau from 1980 to 2005. The person-years of observation were tabulated into one-year classes by age (from 0 to 99+) and calendar time period (from 1980 to 2005) [6,27].

### Cancer cases

Although the Doubs Cancer Registry was established in 1976, only NHL incidence data registered between 1980 and 2005 were included to minimise the risk of under-registration in the early years. With only one tertiary referral hospital in the region (the University Hospital), reporting is homogeneous and complete, as ascertained by the ratio of the number of deaths to the number of cases registered during 1983-1987, which at 47% is very similar to those reported in other Western countries [28]. Virtually all cases were histologically verified (99%). The International Classification of Disease for Oncology, third edition (ICD-O-3), has been used to classify cases since the 2002 diagnosis year, but for cases diagnosed before 2002 and classified according to earlier versions of ICD O, the IARCtools program was used to convert codes to ICD-O-3 [29]. When required by the software, a hand review was performed by a medical expert in cancer registration for complete conversion. The Doubs cancer registry extracted anonymous NHL cases (using the ICD-O-3 morphology codes M9590-9596/3, M9670-9719/3, M9727-9729/3 and 9832-9834/3) and tabulated them into one-year classes by age and calendar time period. The lack of an adequate sample size precluded analyses by histological subtype. The procedures at the Doubs cancer registry were approved by the French National Cancer Registry Committee and the National Commission for the Confidentiality of Computerized Data.

### Statistical analysis

Age-standardised rates were calculated with the World Health Organization (WHO) world population serving as the standard [30].

We restricted the APC analysis to cases aged 20-89 because of too few events in the younger age groups and lower data quality in the oldest age group. To obtain the effects of age, period and cohort, a log-linear model was fitted to the data by assuming a Poisson distribution for the observed number of NHL cases. The general form of the multiplicative APC model for rates, λ(a, p) at age a in period p for persons in cohort c = p - a, is as follows:

log[λ(a,p)]=f(a)+g(p)+h(c),

where a, p and c represent the mean age, period and cohort, respectively, for the observational units; f, g and h are parametric functions.

The "classical" approach to modelling APC effects, in which variables are defined as "factors", uses one parameter per distinct value of age, period and cohort to accommodate the non-linearity of the effects. Because the three variables of age, period and cohort were originally continuous, we modelled these effects by parametric smooth functions. Restricted B-splines (natural splines) with seven parameters for the age, period and cohort terms were incorporated in the APC model to reduce random variation. We arranged the relevant submodels into a sequence that gives all the relevant comparisons between adjacent lines from an analysis of deviance. To test for the significance of effects between nested models, we compared the difference in deviance between these different models using the F test. Statistical significance was attributed to two-sided p-values < 0.05.

To allow reconstruction of the fitted rates from the reported values and to overcome the so-called identifiability problem, we chose the following parameterisation proposed by Carstensen [31]. We had an a priori assumption that mainly period-effects drive the change in rates, considering that the whole population would be equally exposed to environmental risk factors and supported by the fact that risk factors for NHL seem to be unrelated to birth cohort [32]. We therefore fitted models sequentially. First, we fit an age-period model, choosing the first year (1980) as the reference period. Second, the logarithms of the fitted values from this model were used as an offset variable in a model with cohort effects. Third, the cohort effects from this model were used as the residual log rate ratios by cohort. The drift parameter (representing a linear secular trend not exclusively identifiable as a period or cohort effect) was extracted using the weighted average (by marginal number of cases). The age function is interpretable as the log of the age-specific rates for the reference period (i.e., cross-sectional rates). The period function represents the log-rate ratios relative to the reference period, while the cohort function represents the log-rate ratios relative to the age-period prediction (residual log-rate ratios). In examining these functions, minor fluctuations should not lead to direct, thus limited, interpretation; only the major overall trends should be considered.

All data were analysed using the R 2.10.0 statistical software (Epi 1.1.9 package) (R Development Core Team, 2009).

## Results

### Descriptive data

A total of 1,457 incident cases of NHL were registered in the Doubs region between 1980 and 2005 in the age group from 20-89 years, for a corresponding population of 367,842 in 1999 (census data).

**Figure 1:**
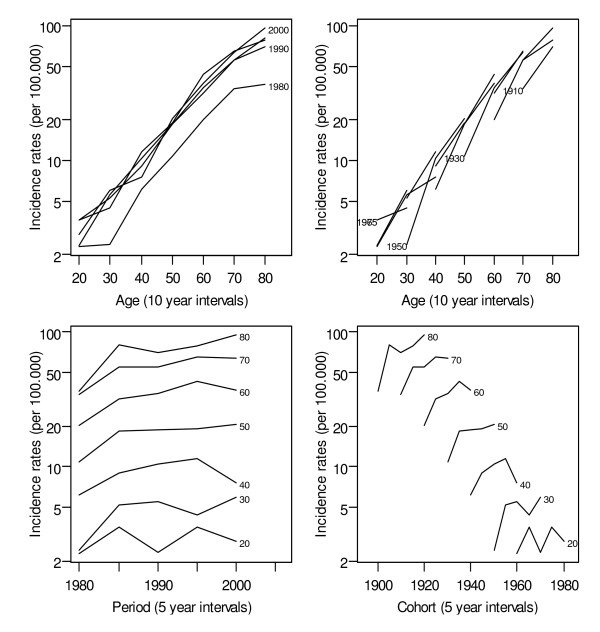
**Incidence of non-Hodgkin's lymphoma per 100,000 person-years by age and birth cohort (age group 20-89 years, 1980-2005, Doubs region, France)**. Top left: Age on the x-axis; the rates corresponding to the same period are connected by lines. Top right: Age on the x-axis; the rates corresponding to the same cohorts are connected by lines. Bottom left: Period on the x-axis; the rates corresponding to the same age groups are connected by lines. Bottom right: Cohort on the x-axis; the rates corresponding to the same age groups are connected by lines.

The age-standardised incidence rate increased from 4.7 in 1980 to 11.9 per 100,000 person-years at risk in 1992 (corresponding to a 2.5-fold increase) and stabilised afterwards (11.1 per 100,000 in 2005). The observed rates, aggregated into 5-year periods and 10-year age classes to produce fairly stable rates, are plotted in Figure 1. A clear gradient of higher NHL incidence rates with increasing age is observed throughout the analysed period. No interaction between age and period is noticeable, as indicated by the parallel lines (on a log scale) in the first and third plots. Age-specific rates are therefore proportional between periods, suggesting an age-period model. There is a clear tendency in the 1980-1984 period that shows lower rates than subsequent periods. No clear pattern emerges from the cohort curves.

### Age-period-cohort analysis

The data supported a development of NHL incidence more complex than a mere linear trend over time. Table 1 shows the changes in deviance in the sequential building of the model. The age-drift model significantly improved the fit over the age-only model (p < 10^-15^). Large period curvature effects, both adjusted for cohort effects and non-adjusted (p < 10^-4 ^and p < 10^-5^, respectively), showed departure from linear periodic trends. In both the APC model and the AC model, cohort curvature effects were not statistically significant (p = 0.46 and p = 0.08, respectively).

**Table 1:** Comparison of age-period-cohort submodels for the incidence of non-Hodgkin's lymphoma to separate contributions from each of the time variables (age group 20-89 years, 1980-2005, Doubs region, France).

Terms in model	D^a ^(df)	Effect	ΔD (Δdf)	p value
Age	1848 (1812)	-	-	-
Age + Drift	1764 (1811)	δ^b^|A	84 (1)	< 10^-15^
Age + Period	1727 (1805)	P^c^|A	37 (6)	< 10^-5^
Age + Period + Cohort	1721 (1799)	C^c^|A, P	6 (6)	0.46
Age + Cohort	1753 (1805)	P^c^|A, C	32 (6)	< 10^-4^
Age + Drift	1764 (1811)	C^c^|A	11 (6)	0.08

^a ^deviance

^b ^drift or linear secular trend

^c ^curvature or non-linear effect

^d ^the submodels are arranged in a sequence that gives all the relevant tests as comparisons between adjacent lines (using the difference in deviances and the F test). The successive tests (all adjusted for age) refer therefore to the drift, the non linear effect of period, the non linear effect of cohort (adjusted for period), the non linear effect of period (adjusted for cohort) and the non linear effect of cohort.

**Figure 2:**
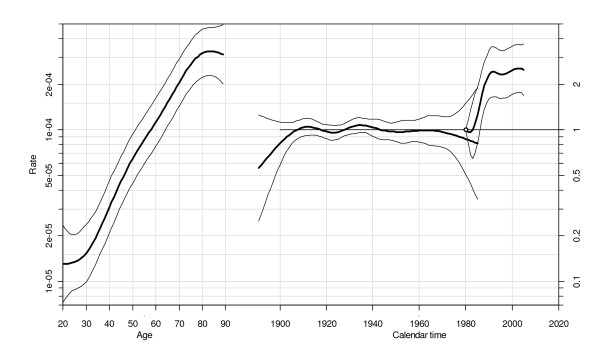
**Estimated effects from the age-period-cohort model (non-Hodgkin's lymphoma, age group 20-89 years, 1980-2005, Doubs region, France)**. The centermost curve represents the age-specific rates for 100,000 person-years at risk during the reference period (1980). The median curve shows the rate ratios of cohorts conditional on the estimated age and period effects. The rightmost curve shows the rate ratios of periods relative to the reference period (1980). Fitted values are plotted together with 95% confidence limits.

The age, period and cohort effects are displayed in Figure 2 on a directly comparable scale, allowing the slopes of the effects to be compared. Age effects showed a steadily increasing slope up to the age of 80, levelling off for older ages. From the period-effect curve, two non linear and thus identifiable changes merit special attention: period effects jumped markedly in 1983 and stabilised in 1992 after a 2.4-fold increase (compared to the 1980 period), which was statistically significant. The conditional cohort rate ratios remained close to one and did not vary significantly (all rate ratio 95% confidence intervals included one, while broadening at the extremes due to low NHL counts).

## Discussion

The detailed tabulation combined with parametric smooth functions allowed relevant features to be captured. The striking observation from this APC analysis concerns the strong 1983-1992 period effect highlighted in the Doubs region. These two inflection points are second-order features and are therefore not an artefact of the parameterisation.

We took advantage of the long tenure of the Doubs registry database to analyse trends in the incidence of NHL over a sufficiently extended time period (26 years). NHL comprises many histologically distinct lymphocyte malignancies, each with putatively distinct aetiologies (and time scale patterns). Unfortunately, in this study, the NHL subtypes were not considered because small case counts precluded the detection of subtle changes in APC effects.

In the Doubs region, age-standardised NHL incidence rates and trends are similar to those reported from other European countries [3,33]. Although changes in the NHL incidence are discussed at length in the medical literature, few studies have attempted to separate the respective contributions of the three time-scales to the observed trends. Using the Clayton-Schiffers method, McNally et al. found no evidence of non-linear period or non-linear cohort effects during the period from 1978-1991 in Yorkshire (UK) [34]. Pollan et al. carried out APC analyses but replaced the period of diagnosis with a variable reflecting the availability of new medical technologies (to avoid the identifiability problem), which is a questionable assumption [9]. They ascribed the increase in incidence (Spain, 1973-1991) to period and cohort effects. Bray et al. used Bayesian APC models (mainly to project NHL incident rates) and concluded there was a mixture of period and cohort effects (without further specification) in nine different countries (1973-1992) [2]. Liu et al. concluded that period effects played a major role in NHL incidence trends between 1970 and 1996 in Canada [34]. Sandin et al. stressed the predominance of calendar period over birth cohort effects in the Nordic countries from 1960 through 2003 [5]. Adamson et al. modelled only the drift [32].

The overall time trend patterns of our study fit with the APC-specific assessments by Sandin et al. and Liu et al. [5,35]. In terms of the interpretation of period effects, three factors may have affected the observed trends: multiple schemes for lymphoma classification, advancements in diagnosis and widely distributed risk factors.

The complex and evolving classification may have had some role particularly between 1995 and 2001. During this period, diagnoses were originally described by pathologists using the WHO classification (an extension of the former Revised European American classification of Lymphoid Neoplasms [REAL]), but diagnoses were coded by cancer registries as ICD-O-2 (incorporating another scheme, the Working Formulation) [33]. Conversely, ICD-O-3, used by cancer registries to classify cases from 2002 onwards, is known to reduce misclassification bias [36,37]. Considering the time lag between the increase in period effects (1983-1992) and the evolving classification (from 1995 onwards), it is unlikely that the latter could explain the former.

An increase in period effects may also relate to the combined effects of improvements in NHL detection (lowering the threshold of detection) and the widespread use of new methods and techniques (allowing greater access to interventions) [35]. In this scenario, NHL incidence rates would be maintained at their current level (due to the full implementation of modern diagnostic procedures), reflecting the totality of disease in a population.

The changes in classification and the improvements of diagnostic accuracy cannot largely account for the steady increases registered in NHL incidence rates up to the late 1990s [3,7], giving way to an increasing exposure to risk factors as the more likely explanation. Because everyone broadly consumes the same food items and shares essentially the same outdoor environment, toxicants present in the food chain or in the environment could influence disease incidence. However, the contribution of other potential risk factors (diet rich in proteins and fats, or medical drugs) cannot be ruled out [35].

The 1983-1992 period-effect increase highlighted in the present study, affecting all ages equally, could be due to one or more exposures that emerged during the 1960s (considering a 20-year latency period), with a wide and increasing use and release into the environment. Agents with immunosuppressive activity, such as persistent organic pollutants (dioxins, chlorophenols and polychlorinated biphenyls) and pesticides (particularly the phenoxyacetic acids) meet these criteria [38,39]. Although exposure to pesticides has mainly been occupational, the situation for persistent organic pollutants is quite different because the whole population was exposed, mainly thorough the food chain (e.g., fatty fish, meat, dairy products). In this respect, the majority of exposure would not come from the Doubs region because most of these food products were not produced locally but rather had been transported over hundreds of kilometres. These suspected widely distributed risk factor first increased and subsequently decreased (because of regulations enforced in France in the 1980s and the 1990s). Thus, if the environmental hypothesis holds, the period effects of NHL incidence (and not only the incidence rates) are expected to start their decline in the near future.

## Conclusions

The increased NHL incidence in the Doubs region is mostly dependent on factors associated with age and calendar periods instead of cohorts. We found evidence for a levelling off in both incidence rates and period effects beginning in 1992. Continued NHL incidence surveillance and careful analysis of period effects are of utmost importance to elucidate the enigmatic epidemiology of NHL.

## Competing interests

The authors declare that they have no competing interests.

## Authors' contributions

JFV designed the study, performed the statistical analyses and drafted the manuscript. EF managed the data and revised the paper. AD supervised data collection and revised the paper. All authors read and approved the final draft.
